# Ballroom dance and balance ability in older adults: a serial mediation model of fear of falling and physical self-efficacy

**DOI:** 10.3389/fspor.2026.1858245

**Published:** 2026-06-12

**Authors:** Dongquan Gao, Xiaojie Sun, Hongpeng Zhou, Donghan Yan, Nur Shakila Mazalan, Denise Koh

**Affiliations:** 1 National University of Malaysia, Bangi, Malaysia; 2 Hebei Chemical and Pharmaceutical College, Shijiazhuang, China; 3 Hebei Vocational College of Rail Transportation, Shijiazhuang, China

**Keywords:** balance ability, ballroom dance, fear of falling, older adults, physical self-efficacy, serial mediation

## Abstract

**Background:**

Global population aging has made the physical and mental health of older adults a critical public health concern. Balance ability is essential for independent living and fall prevention, but the psychological mechanisms linking ballroom dance to balance ability remain understudied. This cross-sectional study explored the serial mediating roles of fear of falling and physical self-efficacy in the association between ballroom dance and balance ability among community-dwelling older adults.

**Methods:**

A cross-sectional design was adopted, recruiting 318 community-dwelling older adults who regularly participated in ballroom dance from northern China. Validated scales (Physical Activity Rating Scale-3, Berg Balance Scale, Fall Efficacy Scale-International, and Physical Self-Efficacy Scale) were used to collect data. Balance ability was assessed in person by trained research assistants using the Berg Balance Scale. Structural equation modeling and the PROCESS macro were applied to test the serial mediation model.

**Results:**

(a) Ballroom dance exercise was positively associated with balance ability in older adults. (b) Fear of falling and physical self-efficacy each played a significant independent mediating role between ballroom dance and balance ability. (c) Fear of falling and physical self-efficacy exerted a significant serial mediating effect, with three valid mediating pathways identified. The individual mediating effect of physical self-efficacy was the strongest among all pathways.

**Conclusion:**

Lower fear of falling and higher physical self-efficacy are associated with better balance ability in older adults who participate in ballroom dance. These findings provide preliminary correlational evidence for community-based fall prevention and healthy aging programs, while causal interpretations require longitudinal validation.

## 1 Introduction

Global population aging has become a major public health challenge, with maintaining physical function—especially balance ability—being critical for older adults' independent living and fall prevention (1). In China, the growing older population has drawn widespread attention to fall risk, as falls are a leading cause of injury, disability, and mortality among community-dwelling older adults (2). Age-related physiological changes (e.g., reduced muscle strength, impaired sensory function) lead to balance decline, increasing fall risk, while fear of falling triggered by balance impairment further reduces physical activity participation, forming a vicious cycle (3, 4).

Leisure physical activity is considered an effective non-pharmacological intervention associated with better balance ability and lower fall risk in older adults (5). Ballroom dance, a popular group activity in China, integrates rhythmic movement, coordination, and social interaction, making it suitable for older adults with varying physical conditions (6). Unlike single, repetitive exercises, its combination of physical movement and music is linked to enhanced physical function while providing emotional and social benefits (7).

Existing studies have shown that regular ballroom dance is associated with better balance ability, muscle strength, and psychological states in older adults (8, 9). However, most research focuses on direct physical effects, ignoring the psychological mechanisms (e.g., fear of falling, physical self-efficacy) underlying the relationship between ballroom dance and balance ability. To fill this gap, this cross-sectional study explored the serial mediating roles of fear of falling and physical self-efficacy, aiming to examine the correlational mechanisms and provide theoretical references for healthy aging and fall prevention interventions.

### 1.1 Ballroom dance and balance ability

Balance ability—the capacity to maintain postural stability and adjust body position in static or dynamic states—reflects muscle strength, sensory integration, and motor coordination (10). Both static and dynamic balance decline with age, with dynamic balance (critical for daily activities like walking and turning) declining more significantly after 60 years (11). Good balance ability is essential for independent living and fall prevention (12).

Ballroom dance is associated with better balance ability through its rhythmic, coordinated movements (e.g., rotation, step changes), which train the vestibular system and proprioception, potentially contributing to dynamic balance (13). Compared with square dancing, walking, or Tai Chi, ballroom dance participants show better dynamic balance performance (14, 15). Its partner-based nature requires continuous postural adjustment, which may strengthen the body's response to balance challenges (16). Additionally, ballroom dance is associated with better lower limb muscle strength and lower osteoporosis risk, further relating to balance ability (17, 18).

### 1.2 Fear of falling and balance ability

Fear of falling—a negative emotional state characterized by anxiety about falling during daily activities—is triggered by fall experiences, balance impairment, or perceived physical decline (19). It includes emotional fear (anxiety) and behavioral avoidance (avoiding high-risk activities) (3), with a prevalence of 30%–50% among adults aged ≥60 years, increasing with age and fall frequency (20). Fear of falling is associated with reduced physical activity participation, which may lead to muscle atrophy and further balance decline, forming a vicious cycle (4).

Ballroom dance, as a low-risk group activity, provides a safe, supportive environment that may be associated with lower fear of falling (20). Social interaction theory suggests that group activities enhance social support and psychological resilience, which may relate to reduced negative emotions like fear of falling (21). Repeated practice of balance-related movements in ballroom dance may build confidence, which could be associated with reduced anxiety and disruption of the vicious cycle between fear of falling and balance decline (22, 23). Thus, fear of falling may mediate the relationship between ballroom dance and balance ability.

### 1.3 Physical self-efficacy and balance ability

Physical self-efficacy—an individual's confidence in completing specific physical activities—is a key psychological factor influencing physical activity behavior and physical function (21). It includes strength, balance, and endurance self-efficacy, all closely linked to balance ability and fall prevention in older adults (3, 24). The WHO emphasizes physical self-efficacy as a core component of healthy aging (3).

Group-based physical activities like ballroom dance are associated with higher physical self-efficacy through skill improvement and social recognition (25). Compared with yoga, ballroom dance has been found to be more effective in improving balance and movement self-efficacy (22). Social cognitive theory notes that ballroom dance provides personal experience, social support, and positive feedback—key factors shaping physical self-efficacy (26). In China, older adults gain a sense of achievement from ballroom dance, which is associated with greater confidence in their balance ability and sustained physical activity participation (5, 27). Thus, physical self-efficacy may mediate the relationship between ballroom dance and balance ability.

### 1.4 The serial multiple mediation mechanism of fear of falling and physical self-efficacy

The cognitive-behavioral model of fear of falling posits that negative cognitive evaluations of fall risk and behavioral avoidance affect physical self-efficacy and physical function (28). Ballroom dance is associated with lower fear of falling and higher physical self-efficacy through social support and a sense of security (3, 9), while lower fear of falling is associated with better self-perception of physical ability, further relating to higher physical self-efficacy (29).

Fear of falling is negatively correlated with physical self-efficacy: it is associated with reduced physical activity participation and may trigger negative self-evaluations of balance ability (28, 30). Ballroom dance, as a low-risk group activity, is associated with reduced behavioral avoidance and negative cognitive evaluations, thereby relating to higher physical self-efficacy (28). Thus, fear of falling and physical self-efficacy may play serial mediating roles between ballroom dance and balance ability.

### 1.5 The present study

**Figure 1:**
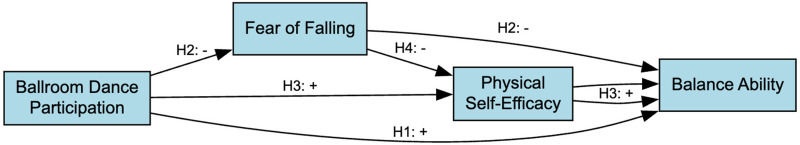
Research hypothesis model. H1, H2, H3, H4 denote the four hypotheses. “+” indicates a positive association, “−” indicates a negative association. The model was tested using cross-sectional data.

Despite growing research on ballroom dance and older adults' health, few studies have explored the serial mediating roles of fear of falling and physical self-efficacy in the relationship between ballroom dance and balance ability (22). This cross-sectional study aimed to fill this gap by examining these mechanisms in a large sample of community-dwelling older adults, providing correlational insights for fall prevention and healthy aging interventions. The following hypotheses were proposed (Figure 1):

H1. Ballroom dance participation is positively associated with balance ability in older adults.

H2. Fear of falling mediates the relationship between ballroom dance participation and balance ability.

H3. Physical self-efficacy mediates the association between ballroom dance participation and balance ability.

H4. Fear of falling and physical self-efficacy serially mediate the relationship between ballroom dance participation and balance ability.

### 1.6 Theoretical framework

This study primarily draws on two core theoretical perspectives: social cognitive theory (26) and the cognitive-behavioral model of fear of falling (31). Social cognitive theory posits that observational learning, social support, and mastery experiences (e.g., successfully performing dance movements) enhance physical self-efficacy, which in turn influences physical function. The cognitive-behavioral model suggests that reducing negative fall-related cognitions alleviates fear of falling, thereby promoting balance-related activities. Other theoretical perspectives (role theory, ecosystem theory, socio-emotional selectivity theory, and social interaction theory) are briefly discussed in Section 4 as complementary explanations for the findings, rather than as *a priori* theoretical framework. This focused approach ensures that the hypotheses are directly derived from testable mechanisms rather than *post hoc* theoretical decoration.

## 2 Materials and methods

### 2.1 Participants

A cross-sectional, questionnaire-based design was used, with in-person balance testing (see 2.2.1). Participants were community-dwelling older adults (aged ≥45 years) from Shijiazhuang, Tangshan, and Handan (northern China) who had participated in ballroom dance for at least 1 month. Inclusion criteria: (1) aged ≥45 years; (2) urban community resident for ≥6 months with stable ballroom dance participation; (3) no cognitive impairment (MMSE score ≥24) to ensure valid self-reporting; (4) no self-reported severe physical diseases affecting balance or ballroom dance participation, specifically including but not limited to: stroke with residual motor impairment, Parkinson's disease, uncontrolled diabetic neuropathy, severe osteoarthritis (Kellgren–Lawrence grade ≥3), or any condition requiring walking aids; (5) voluntary participation and signed informed consent. A total of 23 individuals were excluded based on criterion (4): 8 with uncontrolled diabetic neuropathy, 7 with residual stroke impairment, 5 with Parkinson's disease, and 3 with severe osteoarthritis. Exclusion criteria: (1) incomplete questionnaires (missing ≥10% of items); (2) obvious response bias; (3) incorrect key information reporting; (4) mid-study withdrawal.

Data were collected from September 15 to October 20, 2025, via on-site distribution (community activity centers, ballroom dance venues) and web-based surveys (Questionnaire Star), with IP monitoring to avoid duplicates. All participants completed the self-report questionnaires on-site or online, and those who met inclusion criteria were then scheduled for in-person balance testing (see 2.2.3). Investigators were uniformly trained to standardize questionnaire guidance. A total of 380 questionnaires were distributed; 62 invalid ones were excluded (8 incorrect age, 12 incomplete, 36 response bias, 6 mid-withdrawal), resulting in 318 valid responses (valid rate = 83.68%), meeting cross-sectional study standards (32).

Sample characteristics: Age: 45–59 years (54.1%, *n* = 172), ≥60 years (45.9%, *n* = 146); Gender: Male (22.0%, *n* = 70), Female (78.0%, *n* = 248); Education: Primary school or below (35.2%, *n* = 112), Junior high (42.8%, *n* = 136), Senior high/technical secondary (18.2%, *n* = 58), College or above (3.8%, *n* = 12); Marital status: Married (79.2%, *n* = 252), Widowed (12.6%, *n* = 40), Divorced (5.7%, *n* = 18), Unmarried (2.5%, *n* = 8); Health insurance (94.3%, *n* = 300), Pension insurance (87.4%, *n* = 278); Ballroom dance participation: 2–3 times/week (48.4%, *n* = 154), 4–5 times/week (32.1%, *n* = 102), ≥5 times/week (19.5%, *n* = 62), average duration (8.2 ± 2.5) months.

#### 2.1.1 Ballroom dance context

This study did not implement a structured intervention. All participants were existing ballroom dance practitioners recruited from community settings (e.g., public squares, community activity centers, dance studios) in northern China. Typical ballroom dance sessions lasted 60–90 min, involved partner-based or group-based routines (including waltz, tango, foxtrot, and quickstep), and were guided by volunteer instructors or peer leaders with varying levels of formal training. No standardized choreography, instructor certification, or supervision was imposed across sites. Participants reported dancing 2–5 times per week on average, and all had been practicing for at least one month (mean duration 8.2 ± 2.5 months). Thus, the present study examines cross-sectional associations among existing dancers rather than a controlled intervention.

### 2.2 Measures

- Demographic Information: A self-designed questionnaire based on relevant literature (32) collected sex, age, education, marital status, residency, income, insurance status, and ballroom dance participation (frequency, duration, type). Detailed characteristics are presented in Table 1.
- Ballroom Dance Exercise: Measured by the Physical Activity Rating Scale-3 [PARS-3; (33)], a 3-item scale assessing exercise intensity, duration, and frequency. Total scores range from 3 to 15, with higher scores indicating higher ballroom dance participation. Cronbach's *α* for PARS-3 in this study was 0.652, which is slightly below the conventional threshold of 0.70 (34, 35). Given this marginal internal consistency, we conducted a sensitivity analysis by removing participants in the lowest and highest 10% of PARS-3 scores. The mediation results (direction and significance of all indirect effects) remained stable, suggesting that the lower alpha did not substantively bias the main findings. Nevertheless, this limitation is acknowledged in Section 4.5.
- Balance Ability: Assessed by the Berg Balance Scale [BBS; (36)], a 14-item scale measuring static and dynamic balance. Unlike self-reported measures, the BBS was administered in person by two trained research assistants (senior physiotherapy students) who completed a standardized training session on BBS administration and scoring. Assessments took place at community activity centers in a quiet, well-lit space. Each participant completed all 14 BBS items under standardized conditions (e.g., standing unsupported, sitting to standing, transfers, turning 360 degrees, single-leg stance), with an average administration time of 15–20 min. Assessors were blinded to participants’ ballroom dance frequency and self-reported questionnaire responses. Scores range from 0 to 56, with higher scores indicating better balance. Cronbach's *α* = 0.917 in this study.
- Fear of Falling: Measured by the Fall Efficacy Scale-International [FES-I; (37)], a 16-item scale assessing confidence in avoiding falls during daily activities. Scores range from 16 to 64, with higher scores indicating greater fear of falling. Cronbach's *α* = 0.862 in this study.
- Physical Self-Efficacy: Assessed by the Physical Self-Efficacy Scale [PSES; (38)], a 10-item scale measuring confidence in physical abilities. Scores range from 10 to 40, with higher scores indicating higher physical self-efficacy. Cronbach's *α* = 0.879 in this study.

**Table 1:** Demographic characteristics of the participants (*N* = 318).

Demographic characteristics	Categories	n	Percentage (%)
Gender	Male	70	22.0
	Female	248	78.0
Age (years)	45–59	172	54.1
	≥60	146	45.9
Education level	Primary school or below	112	35.2
	Junior high school	136	42.8
	Senior high school/Technical secondary school	58	18.2
	College or above	12	3.8
Marital status	Married	252	79.2
	Widowed	40	12.6
	Divorced	18	5.7
	Unmarried	8	2.5
Health insurance	Yes	300	94.3
	No	18	5.7
Pension insurance	Yes	278	87.4
	No	40	12.6
Ballroom dance participation frequency	2–3 times/week	154	48.4
	4–5 times/week	102	32.1
	≥5 times/week	62	19.5

*N* = 318. Percentages may not sum to 100% due to rounding. Data are based on self-reported demographic information collected via questionnaire.

### 2.3 Statistical analysis

SPSS 26.0 and PROCESS 4.0 (39) were used for data analysis. Descriptive statistics described sample characteristics; Pearson correlation analysis examined relationships between variables; Harman's one-factor test detected common method bias. For the serial mediation model, we used bootstrap analysis with 5,000 resamples to generate 95% confidence intervals (CIs) for indirect effects. An indirect effect was considered significant if its 95% CI did not contain zero. Following Hayes (39), we report unstandardized coefficients (*B*) for all paths, including total effect (*c*), direct effect (c'), and indirect effects (*a* × *b*). Structural equation modeling was used to verify the model fit. Given the cross-sectional design, all reported effects are correlational and do not imply causation. This limitation is explicitly addressed in the discussion (Section 4.5).

## 3 Results

### 3.1 Preliminary results

A total of 318 community-dwelling older adults who participated in ballroom dance were evaluated in this study. Table 1 presents the demographic characteristics of the sample. As shown in Table 1, the age of participants was mainly over 45 years: those aged 45–59 accounted for 54.1%, and those aged ≥60 accounted for 45.9%. In terms of gender, female participants accounted for 78.0% (*n* = 248) of the total sample, while male participants accounted for 22.0% (*n* = 70), which is consistent with the actual gender distribution of ballroom dance participants among older adults in China (40).

Regarding living arrangements, most participants lived with family members (spouse and/or children), with only 2.52% living alone. For place of residence, 79.25% lived in urban areas, consistent with the research setting. For education level, the majority had junior high school (42.8%) or primary school (35.2%) education, in line with the educational distribution of urban community-dwelling older adults in northern China (20). Regarding insurance, 85.5% had both pension and medical insurance. Married participants comprised 79.2% of the sample. Overall, the demographic characteristics of the sample were consistent with the target population (urban community-dwelling older adults participating in ballroom dance) and had good representativeness.

### 3.2 Common method bias test

When self-reported methods are used to collect data, the issue of common method bias may arise (41). Common method bias was tested using the Harman one-factor test. The results showed that there were 8 factors with an eigenvalue greater than 1, and the total variance explained by the first common factor was 37.62%, which is less than the critical value of 40%. Therefore, there was no significant common method bias in the data of this study, and the data reliability was acceptable (42).

Since the data were self-reported by the participants, outliers may exist due to input errors or recall bias. To minimize the biases typically associated with self-reported data, several methods were implemented. First, we used SPSS 26.0 software for data cleaning, identifying and reviewing outliers and inconsistent data (e.g., abnormal scores of balance ability, fear of falling, and physical self-efficacy) to ensure the dataset's reliability. Outliers and unlikely responses were excluded, enhancing the overall quality of the data. We also employed standardized, validated questionnaires (PARS-3, BBS, FES-I, PSES) to reduce response biases and ensure consistency across participants' answers (17, 29). To address recall bias, we limited the recall period for questions related to ballroom dance participation (e.g., participation frequency and duration were limited to the past 3 months). Additionally, to mitigate social desirability bias, we assured participants of their anonymity and confidentiality, promoting honest and unbiased responses, particularly in sensitive areas such as fear of falling and physical self-efficacy (18). These combined strategies helped ensure the accuracy and integrity of the self-reported data.

### 3.3 Correlation analysis

The mean value, standard deviation, and Pearson correlation analysis of each key variable (ballroom dance exercise, fear of falling, physical self-efficacy, balance ability) are shown in Table 2. The results showed that ballroom dance exercise was significantly positively correlated with balance ability (*r* = 0.428, *p* < 0.01) and physical self-efficacy (*r* = 0.396, *p* < 0.01), and significantly negatively correlated with fear of falling (*r* = −0.352, *p* < 0.01). Fear of falling was significantly negatively correlated with balance ability (*r* = −0.487, *p* < 0.01) and physical self-efficacy (*r* = −0.413, *p* < 0.01). Physical self-efficacy was significantly positively correlated with balance ability (*r* = 0.524, *p* < 0.01). All correlation coefficients were within the range of 0.1–0.6, indicating no serious multicollinearity problem. These results show that the variables had significant correlations consistent with the research hypotheses, which is suitable for further analysis of mediating effects (17, 20).

**Table 2:** Descriptive statistics and Pearson correlation analysis of variables (*N* = 318).

Variables	M ± SD	1	2	3	4
1. Ballroom Dance Exercise	32.68 ± 10.25	1	−0.352**	0.396**	0.428**
2. Fear of Falling	28.75 ± 6.82	−0.352**	1	−0.413**	−0.487**
3. Physical Self-Efficacy	32.46 ± 5.79	0.396**	−0.413**	1	0.524**
4. Balance Ability	42.39 ± 7.15	0.428**	−0.487**	0.524**	1

1 = ballroom dance exercise (PARS-3 score); 2 = fear of falling (FES-I score); 3 = physical self-efficacy (PSES score); 4 = balance ability (BBS score).

** *p* < 0.01.

### 3.4 Testing the mediating effect

The mediation analysis was performed using the PROCESS 4.0 plug-in in SPSS 26.0 software (39). Hierarchical regression models were generated to examine the associations among variables: Model 1 (ballroom dance exercise → balance ability), Model 2 (ballroom dance exercise → fear of falling), Model 3 (ballroom dance exercise and fear of falling → physical self-efficacy), and Model 4 (ballroom dance exercise, fear of falling, and physical self-efficacy → balance ability). The results are analyzed as follows.

In Model 1, ballroom dance exercise showed a significant positive association with balance ability (*β* = 0.428, *p* < 0.001), corresponding to the total effect (c) in the mediation model. In Model 2, ballroom dance exercise showed a significant negative association with fear of falling (*β* = −0.352, *p* < 0.001). In Model 3, ballroom dance exercise showed a significant positive association with physical self-efficacy (*β* = 0.396, *p* < 0.001); additionally, fear of falling showed a significant negative association with physical self-efficacy (*β* = −0.413, *p* < 0.001).

In Model 4, which added the two mediators, ballroom dance exercise remained significantly associated with balance ability (*β* = 0.187, *p* < 0.001), representing the direct effect (*c*′). Fear of falling was significantly negatively associated with balance ability (*β* = −0.215, *p* < 0.001), and physical self-efficacy was significantly positively associated with balance ability (*β* = 0.382, *p* < 0.001). The details of the hierarchical regression results are summarized in Table 3.

**Table 3:** Hierarchical regression results of the serial mediation model (*N* = 318).

Model	Dependent variable	Independent variable	*β*	*t*	*P*	*R* ^2^	*ΔR* ^2^
Model 1	Balance ability	Ballroom dance exercise	0.428	8.963	<0.001	0.183	0.180
Model 2	Fear of falling	Ballroom dance exercise	−0.352	−7.215	<0.001	0.124	0.121
Model 3	Physical self-efficacy	Ballroom dance exercise	0.396	8.152	<0.001	0.298	0.292
		Fear of falling	−0.413	−8.537	<0.001		
Model 4	Balance ability	Ballroom dance exercise	0.187	4.025	<0.001	0.456	0.273
		Fear of falling	−0.215	−4.682	<0.001		
		Physical self-efficacy	0.382	8.357	<0.001		

*β* = standardized regression coefficient; *p* < 0.001 indicates extremely significant association.

The bootstrap method with 5,000 resamples was used to test the significance of indirect effects; a 95% confidence interval (CI) not containing zero indicated a significant indirect effect (39).

The total effect of ballroom dance on balance ability (unstandardized coefficient) was *B* = 0.241, 95% CI [0.189, 0.295], and the direct effect (*c*′) was *B* = 0.187, 95% CI [0.102, 0.272]. Due to potential suppression or scaling differences between standardized and unstandardized estimates, the sum of the direct and total indirect effects does not exactly equal the total effect; therefore, the significance of indirect effects is the primary focus, as recommended by Hayes (39).

The indirect effects via the three pathways are presented in Table 4. All three indirect effects were significant, as their 95% CIs did not contain zero. The indirect effect through physical self-efficacy (*B* = 0.151) was the largest among the three paths. Accordingly, ballroom dance exercise was not only directly associated with balance ability but also indirectly associated through the independent mediating effects of fear of falling and physical self-efficacy, as well as through the serial mediating effect.

**Table 4:** Bootstrap indirect effects of the serial mediation model (*N* = 318, bootstra*p* = 5,000).

Indirect path	Effect (*B*)	95% CI	Significance
Ballroom dance → Fear of falling → Balance ability	0.076	[0.042, 0.115]	Significant
Ballroom dance → Physical Self-efficacy → Balance ability	0.151	[0.108, 0.197]	Significant
Ballroom dance → Fear of falling → Physical self-efficacy → Balance ability	0.056	[0.031, 0.085]	Significant

*B* = unstandardized indirect effect; CI = confidence interval (bias-corrected). All CIs were computed using 5,000 bootstrap resamples. An effect is significant if the 95% CI does not contain zero. Due to the cross-sectional design, all effects are correlational and do not imply causation.

**Figure 2:**
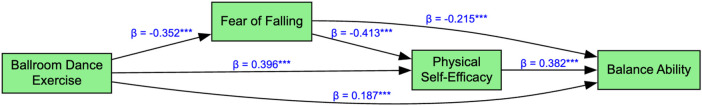
Serial mediation model of ballroom dance exercise, fear of falling, physical self-efficacy, and balance ability. ***p < 0.001. Values are standardized regression coefficients (*β*). All paths were estimated simultaneously using PROCESS macro (Model 6) with 5,000 bootstrap resamples. For clarity, error terms and covariances are not displayed.

The path diagram in Figure 2 shows the standardized regression coefficients (*β*) for each path in the serial mediation model:

1. Ballroom dance exercise was positively associated with balance ability (*β* = 0.187, *p* < 0.001).
2. Ballroom dance exercise was negatively associated with fear of falling (*β* = −0.352, *p* < 0.001).
3. Ballroom dance exercise was positively associated with physical self-efficacy (*β* = 0.396, *p* < 0.001).
4. Fear of falling was negatively associated with balance ability (*β* = −0.215, *p* < 0.001), and physical self-efficacy was positively associated with balance ability (*β* = 0.382, *p* < 0.001).
5. Fear of falling was negatively associated with physical self-efficacy (*β* = −0.413, *p* < 0.001), indicating that lower fear of falling was associated with higher physical self-efficacy.

## 4 Discussion

In this cross-sectional survey of 318 community-dwelling older adults in northern China who participated in ballroom dance, we examined the associations between ballroom dance exercise and balance ability, and the mediating roles of fear of falling and physical self-efficacy. Physical self-efficacy is closely related to physical function, psychological confidence, and motor performance. Characterized by moderate intensity, rhythmic movements, and social interactivity, ballroom dance may comprehensively relate to older adults' physical function and psychological state, and thereby be associated with balance ability. The results support the proposed serial mediation model and are consistent with all research hypotheses (H1–H4), while noting that causal inferences cannot be drawn due to the cross-sectional design.

### 4.1 The direct association between ballroom dance and balance ability

Physical activity (PA) is crucial for maintaining physical function and preventing falls in older adults (43), and it is positively associated with balance ability (29). PA is associated with better balance through maintaining muscle strength, coordination, and flexibility (34), and is linked to higher physical self-efficacy and lower fear of falling, thereby relating to better motor control and postural stability (20). This study found that ballroom dance was positively associated with balance ability (*β* = 0.428, *p* < 0.001), which is consistent with H1.

Social cognitive theory explains this association: older adults' physical self-efficacy and perception of movement safety may directly affect motor behavior and balance control (44). With age, balance tends to decline due to muscle atrophy, reduced joint flexibility, and slower reaction speed (36). In this study, 79.25% of participants were urban residents, who may have better access to sports venues, instructors, and group resources under China's national fitness policies. Urban older adults may have stronger health awareness, and family/friend support may enhance their motivation to participate in ballroom dance, underpinned by recognition of “aging well” (45). Long-term participation fosters stable social relationships, generating positive emotional experiences that, combined with physical benefits, are associated with better balance and successful aging (46). With moderate intensity and low threshold, ballroom dance attracts older adults with different educational levels—77.99% of participants had junior high school education or below, reflecting the preference of this generation for low-barrier exercise and socialization (47).

Participants had an average participation duration of 8.2 ± 2.5 months, with 80.5% participating ≥2 times/week. Regular participation is associated with enhanced core muscle strength, limb coordination, joint flexibility, and cardiorespiratory endurance, which in turn relate to better physical health and balance (27). Ballroom dance's musical rhythm may promote cerebral cortex activity and be associated with better cognitive function and reaction speed (61), while dynamic movements (rotation, step changes) train balance control and vestibular function (36). Rhythmic exercise may also trigger positive emotions, motivating active participation and further relating to better balance (48). In summary, regular ballroom dance is associated with better balance in older adults, which may contribute tolower fall risk and active aging, although causal claims require longitudinal validation.

### 4.2 The mediating role of fear of falling

Fear of falling is critical to older adults' physical health and independent living (37). Ballroom dance was significantly negatively correlated with fear of falling (*r* = −0.352, *p* < 0.01), and it was associated with balance through fear of falling, which is consistent with H2. Fear of falling is a negative psychological state linked to physical function, fall history, and environmental factors (8). Older adults with strong fear of falling tend to avoid balance-related activities, which may lead to further declines in muscle strength and balance, forming a vicious cycle (49). Consistent with previous research (29), higher PA participation is associated with lower fear of falling.

The indirect effect of fear of falling (*B* = 0.076, 95% CI [0.042, 0.115) was significant but smaller than that of physical self-efficacy. Ballroom dance enriches physical activity experience, is associated with better balance control, and relates to lower fear of falling, thereby being associated with better balance (27, 50). This relatively smaller effect may be attributed to three factors: First, 82.71% of participants lived with spouses or children, whose support (e.g., assisting with daily balance-related activities) may reduce fear of falling, aligned with China's traditional filial piety norms (3, 28). Second, 79.25% of participants were urban residents, who may benefit from better public facilities, safer environments, and lower loneliness, all of which are associated with lower fear of falling (20, 51). Third, ballroom dance's group nature provides mutual support and protection, which may reduce fear of falling (19, 52). Participants' moderate fear of falling (*M* = 28.75 ± 6.82), which may reflect no recent fall history, also could have attenuated the mediating effect. In conclusion, fear of falling plays a significant but relatively weaker mediating role in the association between ballroom dance and balance ability.

### 4.3 The mediating role of physical self-efficacy

Physical self-efficacy is an individual's confidence in completing physical activities, involving physical function, movement ability, and psychological confidence (53). Ballroom dance was significantly positively correlated with physical self-efficacy (*r* = 0.396, *p* < 0.01), and physical self-efficacy was positively correlated with balance (*r* = 0.524, *p* < 0.01), supporting H3. Consistent with previous research (38), higher PA is associated with higher physical self-efficacy. Regular ballroom dance is associated with better physical condition and may cultivate physical confidence through movement mastery (3).

The indirect effect of physical self-efficacy (*B* = 0.151, 95% CI [0.108, 0.197) was the largest among the three indirect paths. Long-term ballroom dance is associated with improved joint function, core strength, coordination, and cardiorespiratory endurance, which may reduce muscle and bone loss and enhance balance control (17); these physical changes are directly associated with higher physical self-efficacy (54). However, lack of professional guidance may lead to incorrect postures, which could be associated with lower physical self-efficacy (46). Psychologically, music and social interaction in ballroom dance may reduce anxiety, enhance self-worth, and be associated with higher physical self-efficacy (22, 49), consistent with socio-emotional selectivity theory (55). Notably, Tai Chi and yoga may have more targeted effects on balance and physical self-efficacy (34, 56).

Long-term ballroom dance may form a positive loop: participation → higher physical self-efficacy → better balance (20, 57), transforming participation into positive physical and psychological experiences. Thus, physical self-efficacy plays a key mediating role, representing the strongest indirect pathway in this cross-sectional study.

### 4.4 The serial multiple mediation mechanism of fear of falling and physical self-efficacy

This study examined the serial mediating role of fear of falling and physical self-efficacy, which has rarely been discussed in previous research. Fear of falling was significantly negatively correlated with physical self-efficacy (*r* = −0.413, *p* < 0.01), supporting H4: lower fear of falling is associated with higher physical self-efficacy. PA is associated with enhanced physical capability and confidence, which relates to higher physical self-efficacy and lower fear of falling (53). Social identity theory suggests that ballroom dance's group nature fosters a sense of belonging, linking social interaction to higher physical self-efficacy and lower fear of falling (49, 58).

The serial indirect effect (ballroom dance → fear of falling → physical self-efficacy → balance) was *B* = 0.056, 95% CI [0.031, 0.085], which was smaller than the independent effect of physical self-efficacy but still significant. This smaller magnitude may be attributed to the sample characteristics: 79.25% of participants were urban residents, who may have better healthcare, social welfare, and fitness opportunities, which are associated with lower fear of falling and higher physical self-efficacy (2, 49). The sample's relatively young age (mean 54.3 ± 7.8 years) and likely absence of fall history may also have reduced the chain effect, as did the relatively weaker independent mediating effect of fear of falling.

Similar dance interventions (e.g., modified dance) provide support for this serial mechanism (59), though small sample sizes limit generalizability. In conclusion, ballroom dance is associated with lower fear of falling and higher physical self-efficacy, which in turn are associated with better balance. Future research should compare urban/rural and different age groups to explore contextual differences.

### 4.5 Limitations and future research directions

This study has several limitations that should be considered when interpreting the findings.

First, the cross-sectional design precludes causal inferences. The observed associations may be subject to reverse causality (e.g., better balance → more dance participation) or unmeasured third variables (e.g., baseline fitness, genetics, personality traits). Longitudinal studies (e.g., 12-month tracking) and randomized controlled trials are needed to establish temporal order and causality (20, 59).

Second, the sample lacks generalizability. Participants were predominantly female (78.0%) and urban residents (79.2%) from northern China, limiting generalizability to male, rural, or southern Chinese older adults, as well as to non-ballroom-dance participants. Findings should therefore be interpreted as preliminary evidence for urban-dwelling older women in northern China.

Third, important confounders were not measured. Prior fall history—a strong predictor of fear of falling and balance—was not collected. Additionally, chronic conditions affecting balance (e.g., peripheral neuropathy, vision impairment, vestibular disorders) were only excluded via self-report without systematic assessment or specific prevalence reporting. Future studies should include objective measures of fall history and medical comorbidities.

Fourth, the marginal internal consistency of the PARS-3 (*α* = 0.652) may have attenuated observed associations. Although sensitivity analyses confirmed the stability of the mediation results, this measurement limitation should be acknowledged. Future research should use dance-specific or more reliable physical activity measures.

Fifth, despite efforts to minimize social desirability and recall bias (e.g., anonymity, limited recall period), self-report bias may remain, particularly for sensitive constructs such as fear of falling.

Sixth, this study examined existing dancers rather than a controlled intervention, so no conclusions about the effectiveness of ballroom dance as an intervention can be drawn.

Finally, the results are specific to the Chinese cultural context; cross-cultural studies are needed to explore whether similar mechanisms operate in other countries. Future research should also include additional variables (e.g., education, chronic diseases, anxiety, exercise motivation, dance frequency/intensity) and examine potential moderators (e.g., gender, age group, urban vs. rural residence).

### 4.6 Implications

Theoretically, this study primarily draws on social cognitive theory (26) and the cognitive-behavioral model of fear of falling (31) to explore the serial mediation model. Other theoretical perspectives (role theory, ecosystem theory, socio-emotional selectivity theory, social interaction theory) serve as complementary explanations for the findings rather than as *a priori* tested frameworks. The study enriches research on ballroom dance and active aging by highlighting psychological mechanisms.

Practically, ballroom dance participation is associated with higher physical self-efficacy, lower fear of falling, and better balance in this sample. While causal claims cannot be made, these correlational findings suggest that promoting ballroom dance in urban communities may be a viable component of fall prevention and healthy aging programs. Communities should strengthen venue construction, provide professional instructors, and offer targeted training. Family support and national fitness promotion of ballroom dance could further support older adults' quality of life. However, intervention studies are needed to confirm whether ballroom dance causally improves these outcomes.

## 5 Conclusion

Based on a cross-sectional study of 318 community-dwelling older adults participating in ballroom dance, this study examined the associations between ballroom dance and balance ability, focusing on the mediating roles of fear of falling and physical self-efficacy. First, ballroom dance was positively associated with balance ability, consistent with previous research (17). Second, fear of falling and physical self-efficacy significantly mediated the association through three pathways: ballroom dance → fear of falling → balance; ballroom dance → physical self-efficacy → balance; and ballroom dance → fear of falling → physical self-efficacy → balance. In this sample, the indirect effect through physical self-efficacy (*B* = 0.151) was larger than that through fear of falling (*B* = 0.076), and the serial indirect effect (*B* = 0.056) was also significant. These findings align with social cognitive theory (53).

These cross-sectional findings suggest that lower fear of falling and higher physical self-efficacy is associated with better balance ability in older adults who participate in ballroom dance. The observed associations—including the potential for ballroom dance to be linked to enhanced physical function, reduced negative psychological perceptions, and better balance—merit further investigation through longitudinal and experimental designs (48). While causal claims cannot be made, this study provides correlational evidence that may inform efforts to support older adults' physical health, independent living, fall risk reduction (51), and China's active aging strategy (60). Future research should employ longitudinal or intervention designs to test whether ballroom dance causally improves balance via these psychological mechanisms.

## Data Availability

The original contributions presented in the study are included in the article/Supplementary Material, further inquiries can be directed to the corresponding authors.
